# Bacteria Localization and Chorion Thinning among Preterm Premature Rupture of Membranes

**DOI:** 10.1371/journal.pone.0083338

**Published:** 2014-01-08

**Authors:** Kimberly B. Fortner, Chad A. Grotegut, Carla E. Ransom, Rex C. Bentley, Liping Feng, Lan Lan, R. Phillips Heine, Patrick C. Seed, Amy P. Murtha

**Affiliations:** 1 Division Maternal-Fetal Medicine, Department of Obstetrics and Gynecology, Vanderbilt University Medical Center, Vanderbilt University, Nashville, Tennessee, United States of America; 2 Division Maternal-Fetal Medicine, Department of Obstetrics and Gynecology, Duke University Medical Center, Duke University, Durham, North Carolina, United States of America; 3 Department of Pathology, Duke University Hospital, Duke University, Durham, North Carolina, United States of America; 4 Division of Infectious Diseases, Department of Pediatrics, Duke University Hospital and Department of Molecular Genetics and Microbiology, Duke University, Durham, North Carolina, United States of America; 5 Department of Biostatistics, Duke University Hospital, Duke University, Durham, North Carolina, United States of America; Fudan University, China

## Abstract

**Objective:**

Bacterial colonization of the fetal membranes and its role in pathogenesis of membrane rupture is poorly understood. Prior retrospective work revealed chorion layer thinning in preterm premature rupture of membranes (PPROM) subjects. Our objective was to prospectively examine fetal membrane chorion thinning and to correlate to bacterial presence in PPROM, preterm, and term subjects.

**Study Design:**

Paired membrane samples (membrane rupture and membrane distant) were prospectively collected from: PPROM = 14, preterm labor (PTL = 8), preterm no labor (PTNL = 8), term labor (TL = 10), and term no labor (TNL = 8), subjects. Sections were probed with cytokeratin to identify fetal trophoblast layer of the chorion using immunohistochemistry. Fluorescence *in situ* hybridization was performed using broad range 16 s ribosomal RNA probe. Images were evaluated, chorion and choriodecidua were measured, and bacterial fluorescence scored. Chorion thinning and bacterial presence were compared among and between groups using Student's t-test, linear mixed effect model, and Poisson regression model (SAS Cary, NC).

**Results:**

In all groups, the fetal chorion cellular layer was thinner at rupture compared to distant site (147.2 vs. 253.7 µm, p<0.0001). Further, chorion thinning was greatest among PPROM subjects compared to all other groups combined, regardless of site sampled [PPROM(114.9) vs. PTL(246.0) vs. PTNL(200.8) vs. TL(217.9) vs. TNL(246.5)]. Bacteria counts were highest among PPROM subjects compared to all other groups regardless of site sampled or histologic infection [PPROM(31) vs. PTL(9) vs. PTNL(7) vs. TL(7) vs. TNL(6)]. Among all subjects at both sites, bacterial counts were inversely correlated with chorion thinning, even excluding histologic chorioamnionitis (p<0.0001 and p = 0.05).

**Conclusions:**

Fetal chorion was uniformly thinner at rupture site compared to distant sites. In PPROM fetal chorion, we demonstrated pronounced global thinning. Although cause or consequence is uncertain, bacterial presence is greatest and inversely correlated with chorion thinning among PPROM subjects.

## Introduction

Complications of preterm birth are complex, costly, and not limited to birth. Mothers of preterm infants have increased rates of: postpartum depression and operative delivery, with longer hospital stays. Neonatal consequences are both immediate, such as respiratory distress [1], necrotizing enterocolitis, and feeding difficulties as well as delayed: childhood challenges with behavior learning [2], [3], motor [4] and visual impairment [5], chronic lung diseases [2], [3], [4], [5], [6], [7] and higher rates of infertility in adult survivors [8]. Nearly one-third of all deliveries occurring preterm are associated with preterm premature rupture of membranes (PPROM) [9]. The need for an improved understanding of the factors initiating preterm membrane rupture is emphasized by the stable rate of preterm births over the last decade [10]. Improved understanding of PPROM will lead to not only more successful treatment regimens, but ultimately to prevention strategies.

Women admitted with PPROM may develop clinical infection (chorioamnionitis), but many do not. Although patients do not have evidence of clinical infection, bacteria can be found in their amniotic fluid and serum inflammatory markers may be elevated [11]. The difference between pathologic bacterial presence (infection) versus symbiotic bacterial presence is not well understood. Further, the correlation between bacterial presence and PPROM is not clear. It is likely that the ability to distinguish between intra-amniotic infection and intra-amniotic inflammation may be due to lack of methodology sensitive enough to detect organisms that are difficult to cultivate or are present in small quantities [12]. Newer molecular techniques have improved the detection of fastidious organisms, such as *Mycoplasma* and *Ureaplasma* that may be pathogenic in fetal membranes [13], [14], [15]. More recent studies have employed bacterial 16 s ribosomal sequencing to identify living and nonliving bacteria across broad taxonomic groups. [16], [17], [18]. However, the impact of microbial invasion on fetal membrane architecture and integrity remains unclear in the pathogenesis of PPROM and deserves further study.

Fetal membrane integrity plays an important role in maintenance of pregnancy throughout gestation. The fetal membranes are primarily composed of two layers, both derived from fetal tissue. The amnion is the innermost layer made up of a single layer of cuboidal epithelial cells and collagen [19]. It provides the majority of tensile strength while in direct contact with the amniotic fluid. The chorion is a thicker outer layer with reticular and trophoblast cells and is in direct contact with maternal decidua[19]. The chorion cell layer is metabolically active [20] and serves a protective role in the maintenance of pregnancy via defense against infection and regulation of apoptosis [21]. It has been demonstrated that the intact assembled fetal membrane, the amnion and choriodecidua, is stronger than its individual components [22]. Also, recent evidence demonstrates variation within the fetal membranes, at least at term, with a “zone of weakness” overlying the lower uterine pole and cervix [23].

Prior work in our lab has demonstrated preferential cell death within the chorion of fetal membranes with premature activation of cellular apoptotic pathways when infection is present [24], [25]. In term subjects, apoptotic cell death was greater with histologic chorioamnionitis [25]. Further, the greatest degree of chorion thinning and apoptotic cell death was found among subjects with PPROM where rates of chorioamnionitis are highest. An unexpected finding in this study was the absence of identifiable chorion layer in nearly 37% of subjects [24]. Specifically, in retrospectively collected membranes, the trophoblast layer was thinner among women with PPROM compared to women delivering preterm without labor and those delivering at term [26]. These findings led to this prospective investigation of fetal membranes from PPROM subjects and their controls.

Given the high rates of chorioamnionitis and the proposed role of infection in the etiology of PPROM, evaluating both bacterial presence and chorion layer thickness will advance our understanding of the pathogenesis leading to premature membrane rupture. We therefore sought to identify patterns of bacterial invasion in PPROM subjects compared to other clinical phenotypes, with the goal of better understanding mechanisms of preterm membrane rupture. Our central hypothesis was that bacteria invade and localize within or adjacent to the chorion layer of the fetal membrane rupture site among women with PPROM, and that this bacterial presence leads to cellular responses with subsequent tissue remodeling, and thinning of the membranes.

## Materials and Methods

### Ethics Statement

This project was approved by the Duke University Institutional Review Board. Women delivering at Duke University Medical Center who completed written consent forms were prospectively enrolled in this observational cohort study.

### Participants

A total of 48 participants, divided by gestational age and exposure to labor, were studied. Fourteen subjects with PPROM were enrolled with gestational ages between 24 and 34 weeks following confirmation of rupture of membranes by physical exam, nitrazine pH test, vaginal pooling, and fern test. They were compared to a cohort of 16 preterm subjects between 24 and 34 weeks of gestation with and without exposure to labor, “preterm labor” (PTL) and “preterm no labor” (PTNL). The PTL (n = 8) subjects were women admitted in active preterm labor, with delivery, regardless of ultimate route of delivery, meeting inclusion criteria. PTNL (n = 8) subjects were delivered by cesarean section without labor for maternal or fetal indications, such as preeclampsia or fetal growth restriction. PPROM and preterm subjects were also compared to 18 term cohorts (37weeks to 41 weeks) with and without exposure to labor, “term labor” (TL) (n = 10) and “term no labor” (TNL) (n = 8). The term labor (TL) group included subjects with gestational age greater than 37 weeks, admitted in labor, regardless of ultimate route of delivery. The term no labor (TNL) group comprised women with intact membranes, without the onset of labor, who delivered via repeat cesarean delivery at greater than 37 weeks.

Gestational ages were based on menstrual dates if the first day of the last menstrual period was concordant with ultrasound, or were based on ultrasound if dates were discordant. Subjects were excluded for major congenital malformation, multiple gestation, or lack of complete specimens collected.

### Tissue collection

A total of 96 paired membrane samples were collected, two samples for each woman enrolled. Following delivery, the fetal membranes were examined for site of rupture, and one strip of membrane was collected and labeled, “rupture site”, readily identified in PPROM, PTL, and TL subjects. In the case of nonlabored subjects with membranes intact, an area overlying the cervix was identified and membranes were marked. All non-labored membranes were inked by applying sterile brilliant green dye to the area of membranes overlying the cervix immediately following delivery of the infant. A second membrane strip was collected from an area distant to rupture site, “distant site”, most commonly a segment of membranes proximate to the placenta (Figure S1). The membrane strip was rolled, sectioned, formalin fixed, and paraffin embedded. Tissue blocks were cut into 3–5 µm sections and mounted on slides. Thus, each slide contained four rolled membrane sections that were sampled from each of the membrane strips. One slide from each site was stained with hematoxylin & eosin and examined for evidence of histologic chorioamnionitis by a single gynecologic pathologist, blinded to the results of the study (RCB). Histologic chorioamnionitis was diagnosed using standardized diagnostic criteria put forth by Redline et al [27].

### Immunohistochemistry

Slides were dewaxed, rehydrated, and prepared for staining with protein digestion using heat, EDTA, and proteinase K. The trophoblast layer within the fetal chorion layer was identified through immunohistochemical staining using a monoclonal antibody directed against cytokeratin (*Cytokeratin MNF-116*, Dako North America, Inc.). Staining cytokeratin allowed recognition of all trophoblast cells: cuboidal epithelial cells in the amnion and metabolically active trophoblast cells in the chorion [20]. Control slides included those stained without primary antibody.

Using the Zeiss Axio Imager at the Duke University Light Microscopy core facility, 2 slides per subject were viewed (rupture site and distant site). Each slide held sections from the four membrane rolls with stained trophoblast layer of the chorion and cuboidal cells of the amnion, as shown in Figure S2. At 10× magnification, images were obtained from four separate areas on each membrane roll. With 4 images per roll, a minimum of 16 digital images per slide or 32 digital images per subject were captured (4 images per roll- one from each quadrant, 4 membrane rolls per slide, and 2 slides per subject). As an example, two sample images of membrane quadrants are depicted in Figure S3.

Using ImageJ® software (*NIH*), each image was reviewed. The thickness of the fetal chorion and choriodecidua were measured. Specifically, the thickness of the trophoblast layer of the fetal chorion was measured; whereas, the reticular layer of the fetal chorion was not included given lack of identifying marker among our samples. Hereafter, we refer to the measured trophoblast portion simply as “chorion.” Secondly, the fused choriodecidua that was measured, included the stained trophoblast layer of the chorion plus adherent decidua. Given inherent membrane variability, tissue processing changes, and variable representative sections, particularly the adherent decidua, measurements of the chorion and choriodecidua were obtained in 4 distinct regions of each image. This protocol yielded a minimum of 128 measurements per slide or 256 per subject. All measurements were performed twice, by the same investigator who was blinded to the specimen's clinical group.

### Fluorescence in situ Hybridization (FISH)

FISH was used to demonstrate the presence of bacteria among these same sections of fetal membrane (96 membrane samples from 48 subjects) using methods previously described by Steele et al [14]. Briefly, paraffin-embedded fetal membrane rolls were dewaxed and rehydrated. Slides were treated with proteinase K digestion to remove proteins that would prohibit probe hybridization. Slides were rinsed, fixed with 0.4% buffered formalin, and then hybridized with the broad range 16 s generic bacterial ribosomal probe in buffer solution overnight at 37°C. A custom oligonucleotide probe was obtained from Invitrogen™ (Life Technologies, Grand Island, NY) with the sequence 5′-F-ACTGCTGCCTCCCGTAGGAGTTTATTCCTT which corresponds with the broad range 16 s ribosomal bacterial subunit. This probe was labeled with Alexa red and was used for FISH [13], [14]. Negative control slides received hybridization buffer without probe. The slides were rinsed, DAPI stained for non-specific identification of nuclei, and mounted with Vectashield® (Vector Laboratories, Inc).

Slides were evaluated under appropriate UV wavelength with the Zeiss Axio Observer at 10X magnification. Images were captured via digital photography using three wavelengths for three color channels: red (probe), blue (DAPI), and green (autofluorescence) (Figure S4). A composite overlay image was obtained by combining the three channels resulting in nuclei appearing blue, bacteria appearing red, and background tissue autofluorescence appearing yellow (red plus green) or green. Any red fluorescence identified on the slide surface was disregarded as contamination. Images were taken from each quadrant per membrane roll, with average of 4 rolls per slide, 2 slides per subject (16 areas examined per slide; 32 per patient). De-identified images were viewed as enlarged digital images on core facility computers (Figure S4) and scored by two examiners for quantity and location of probe fluorescence per membrane quadrant.

### Statistical analysis

Linear mixed effect model was used to assess the clinical group difference in chorion thickness by considering the random-effect caused by the factor of subject, roll, and quadrant. The Poisson regression model with repeated measurement analysis was applied to assess the group difference in total number of bacteria presence. The interaction effect of clinical group and site in the models was tested; the group difference was studied and tested by site if the interaction effect was significant. The predicted chorion thickness and the predicted count of total bacteria at each clinical group were reported using least square means. The comparison of bacteria visualized in the amnion compared to chorion was done as descriptive analysis using least square means in ratio. Statistical analysis was performed with the SAS statistical software (Cary, NC).

## Results

A total of 48 participants, divided by gestational age and exposure to labor, were studied. Fourteen subjects with PPROM were compared to 16 preterm subjects with and without labor exposure and eighteen term subjects with and without labor exposure. Relevant characteristics of the cohort were: similar mean gestational ages for PPROM, PTL, and PTNL groups as well as for the TL and TNL groups. Cesarean delivery rates were higher among PTL group as compared to TL group. The rate of histologic chorioamnionitis was highest among the PPROM and spontaneous PTL group (Table 1). Of the PPROM subjects, the mean latency from rupture of membranes to delivery was 13.1 days. The mean interval from completion of latency antibiotics [28] to delivery was 5.8 days. Eighty-five percent of subjects received ampicillin and azithromycin for a mean of 7 days for PPROM latency; two subjects received an alternate regimen given penicillin allergy. Half of PPROM subjects had at least one episode of vaginal spotting or bleeding.

**Table 1:** Patient Clinical Characteristics.

	PPROM	PTL	PTNL	TL	TNL
	N = 14	N = 8	N = 8	N = 10	N = 8
**Age** (Mean years, (SD))	29.9(5.5)	31.3(6.4)	29.4(6.0)	25.6(5.5)	28.9 (5.7)
**Gestational Age at Delivery** (SD)	29.6(2.6)	30.7(2.8)	30.8(3.6)	38.8(3.4)	39.0(1.2)
**Cesarean Delivery** (percent)	55%	62%	100%	37.5%	100%
**Birthweight** (Mean grams, (SD))	1358(554)	1631(619)	1456(702)	3282(342)	3302(722)
**Chorioamnionitis** (percent)	50%	25%	0%	0%	0%

Clinical maternal and birth characteristics compared by subject enrollment group.

PPROM =  Preterm, premature rupture of membranes (subjects between 24 and 34 weeks following confirmation of rupture of membranes by physical exam).

PTL =  Preterm Labor (subjects between 24 and 34 weeks of gestation with exposure to labor).

PTNL =  Preterm, No Labor (subjects between 24 and 34 weeks of gestation without exposure to labor).

TL =  Term Labor (subjects between 37 and 41 weeks with exposure to labor).

TNL =  Term, No Labor (subjects between 37 and 41 weeks without exposure to labor).

### Immunohistochemistry

**Figure 1:**
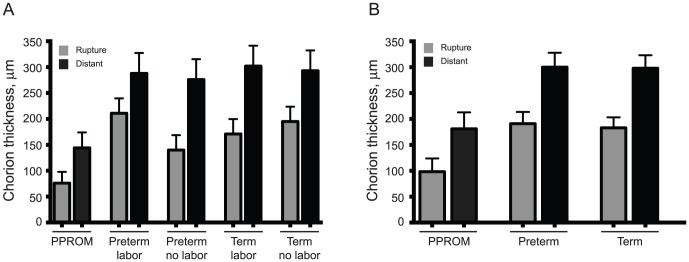
Chorion Thickness by Clinical Group and Gestational Age at Membrane Rupture Site and Distant Site. Figure 1A represents the least square mean chorion thickness among subjects with PPROM as compared to preterm and term cohorts with and without exposure to labor at two distinct membrane sites. Each human fetal membrane was sampled at the site of rupture, “rupture”, and a site distant to rupture, “distant”. Figure 1B includes only subjects without evidence of histologic chorioamnionitis and demonstrates least square mean among PPROM subjects as compared to all other preterm and term subjects. Number reported represents least square mean of minimum of 64 measurements per subject per site sampled. Error bars represent standard errors for each.

Among all subjects enrolled (n = 48), mean chorion thickness measured at any rupture site was significantly less than it's paired distant site, (147.2 µm vs. 253.7 µm, p<0.0001). When mean chorion measurements were compared by clinical group, the chorion layer was thinnest among PPROM subjects compared to preterm and term cohorts with and without labor at both sites sampled (Table 2). Measured chorion at both rupture and distant site was significantly thinner in women with PPROM when compared to all other preterm and term subjects (Figure 1).

**Table 2:** Least Square Means (LS Mean) of Chorion Thickness by Clinical Group and Membrane Collection Site.

	N	Rupture (LS Mean/SE in µm)	P value	N	Distant (LS Mean/SE in µm)	P value
PPROM	14	**76.1 (21.7)**	<0.01*	14	**147.5 (29.7)**	<0.01*
Preterm Labor	8	211.0 (28.7)		8	287.5 (39.3)	
Preterm, No Labor	8	140.4 (28.7)		8	275.9 (39.3)	
Term Labor	10	170.7 (28.7)		10	302.3 (39.3)	
Term No Labor	8	195.2 (28.7)		8	293.1 (39.3)	

Value shown is the least square mean of chorion thickness among all subjects within each of the five clinical groups compared with standard errors. P less than 0.01 by Poisson regression analysis.

*Statistical comparison between PPROM and all other clinical groups (LS Mean).

PPROM =  Preterm, premature rupture of membranes (subjects between 24 and 34 weeks following confirmation of rupture of membranes by physical exam).

Preterm Labor  =  subjects between 24 and 34 weeks of gestation with spontaneous preterm labor.

Preterm, No Labor  =  subjects between 24 and 34 weeks of gestation delivered preterm without labor exposure.

Term  =  subjects between 37 and 41 weeks with labor.

Term, No Labor  =  subjects between 37 and 41 weeks without exposure to labor.

Rupture  =  sample of membrane collected at site of rupture.

Distant  =  sample of membrane collected from an area distant to rupture site, most commonly near the placenta.

The impact of labor on chorion thinning was evaluated. No significant difference in fetal chorion thickness was identified when comparing preterm subjects without membrane rupture (PTL vs. PTNL). This finding persisted at both sites sampled. Likewise, there was no significant difference in fetal chorion thickness among term subjects (TL vs. TNL) at both rupture and distant sites. Table 3.

**Table 3:** Least Square Means (LS Mean) of Chorion Thickness by Gestational Age and Membrane Collection Site.

	Rupture (LS Mean/SE in µm)			Distant (LS Mean/SE in µm)		
	*Labor*	*No Labor*	P value	*Labor*	*No Labor*	P value
Preterm	211.0 (28.7)	140.4 (28.7)	0.08	287.5 (39.3)	275.9 (39.3)	0.83
Term	170.7 (28.7)	195.2 (28.7)	0.55	302.3 (39.3)	293.1 (39.3)	0.87

Value shown is the least square mean of chorion thickness with p values reflecting statistical comparison of chorion between all preterm or term subjects when compared by exposure to labor. P value not significant.

Statistical comparison among gestational ages.

PPROM =  Preterm, premature rupture of membranes (subjects between 24 and 34 weeks following confirmation of rupture of membranes by physical exam)

Preterm Labor  =  subjects between 24 and 34 weeks of gestation with spontaneous preterm labor.

Preterm, No Labor  =  subjects between 24 and 34 weeks of gestation delivered preterm without labor exposure.

Term  =  subjects between 37 and 41 weeks with labor.

Term, No Labor  =  subjects between 37 and 41 weeks without exposure to labor.

Rupture  =  sample of membrane collected at site of rupture.

Distant  =  sample of membrane collected from an area distant to rupture site, most commonly near the placenta.

Recognizing the impact infection may have on cellular remodeling, we repeated the analysis excluding subjects with histologic chorioamnionitis. In this analysis, because no differences were noted among preterm and term subjects in regard to labor exposure, subjects were grouped as Preterm and Term. Evaluation of chorion thickness among PPROM subjects when compared to Preterm and Term subjects after removing those with histologic chorioamnionitis (n = 40) revealed similar results. The fetal chorion was significantly thinner among PPROM subjects as compared to preterm and term subjects even in the absence of histologic chorioamnionitis (Table 4; Figure 1B). Interestingly, this finding existed at both the rupture site and the distant site.

**Table 4:** Least Square Means (LS Mean) of Chorion Thickness by Gestational Age, excluding Histologic Chorioamnionitis, where all preterm and term subjects have been grouped and compared to PPROM subjects once all samples with histologic chorioamnionitis have been excluded.

	Rupture (LS Mean/SE in µm)	P value	Distant (LS Mean/SE in µm	P value
PPROM (n = 10)	**98.3 (25.6)**	**≤0.01**	**181.3 (31.8)**	**≤0.004**
Preterm (n = 12)	191.0 (22.5)		299.7 (27.9)	
Term (n = 18)	183.0 (20.2)		297.7 (25.1)	

P less than 0.01 at the Rupture site; P less than 0.004 at the Distant site.

PPROM =  Preterm, premature rupture of membranes (subjects between 24 and 34 weeks following confirmation of rupture of membranes by physical exam).

Preterm Labor  =  subjects between 24 and 34 weeks of gestation with spontaneous preterm labor.

Preterm, No Labor  =  subjects between 24 and 34 weeks of gestation delivered preterm without labor exposure.

Term  =  subjects between 37 and 41 weeks with labor.

Term, No Labor  =  subjects between 37 and 41 weeks without exposure to labor.

Rupture  =  sample of membrane collected at site of rupture.

Distant  =  sample of membrane collected from an area distant to rupture site, most commonly near the placenta.

To understand whether the entire membrane structure undergoes change in PPROM or if the observed changes are specifically occurring in the chorion layer, we examined the contribution of the chorion as a proportion of total choriodecidua thickness. The results from mixed effect model analysis demonstrate the proportion of chorion/choriodecidua in PPROM subjects is significantly less than in preterm and term groups regardless of site sampled (each p<0.02, Table 5). These data indicate that the fetal chorion layer is the portion of the membrane demonstrating change.

**Table 5:** Proportion Chorion ( =  chorion (µm)/choriodecidua (µm)) by Clinical Group and Membrane Location, where “rupture” is membrane rupture site and “distant” is distal site sampled.

Group	Rupture (Least Square Mean/SE in µm)	Distant (Least Square Mean/SE in µm)
PPROM	**0.19 (0.037)**	**0.27 (0.038)**
Preterm	0.34 (0.035)	0.40 (0.035)
Term	0.36 (0.035)	0.42 (0.036)

Value shown represents the proportion of mean chorion thickness as it relates to total mean choriodecidua thickness with standard errors. Each p<0.0001.

PPROM =  Preterm, premature rupture of membranes (subjects between 24 and 34 weeks following confirmation of rupture of membranes by physical exam).

Preterm  =  subjects between 24 and 34 weeks of gestation with and without exposure to labor.

Term  =  subjects between 37 and 41 weeks with and without exposure to labor.

Rupture  =  sample of membrane collected at site of rupture.

Distant  =  sample of membrane collected from an area distant to rupture site, most commonly near the placenta.

### FISH for Bacterial Localization

Examining all subjects, the mean bacteria count at the rupture site was higher compared to the distant site (18.3 vs. 13.0 bacteria per quadrant, p = 0.0001).

**Figure 2:**
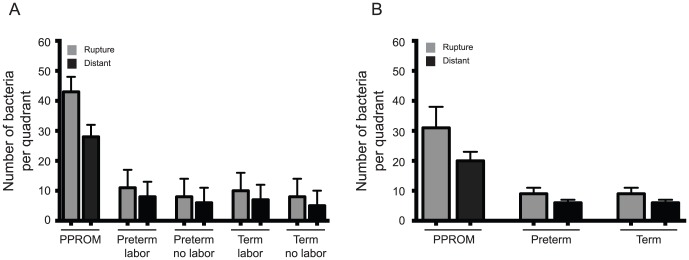
Mean Number of Total Bacteria by Clinical Group and Gestational Age at Membrane Rupture Site and Distant Site. Figure 2A represents the least square mean number of bacteria identified among subjects with PPROM as compared to preterm and term cohorts with and without exposure to labor at “rupture” and “distant” sites. Figure 2B includes only subjects without evidence of histologic chorioamnionitis and demonstrates the least square mean number of bacteria identified among PPROM subjects, compared to all other preterm and term subjects. Number reported represents nearest whole number of the least square mean bacteria identified per membrane quadrant, with 16 sites examined per slide per subject. Error bars represent standard errors for each.

More bacteria were identified among PPROM subjects compared to all other subjects enrolled. At both membrane sites examined, PPROM subjects had greater mean bacterial counts compared to their preterm and term cohorts (Table 6; Figure 2A). Further, the number of bacteria identified in fetal membranes among all preterm subjects was similar, indicating no impact from the process of labor or vaginal passage. Likewise, term subjects had similar bacterial counts among those with and without labor, and likewise were grouped. All specimens had bacteria present, regardless of membrane rupture or labor status.

**Table 6:** Number of Total Bacteria by Clinical Group and Membrane Location.

	N	Rupture Site LS mean (SE)	P value	N	Distant Site LS mean (SE)	P value
PPROM	**14**	**43 (5)**	**<0.0001**	**14**	**28 (4)**	**<0.003**
Preterm Labor	8	11 (6)		8	8 (5)	
Preterm, No Labor	8	8 (6)		8	6 (5)	
Term Labor	10	10 (6)		10	7 (5)	
Term, No Labor	8	8 (6)		8	5 (5)	

Value shown represents the mean number of bacteria visualized at each membrane location among all subjects within each of the five clinical groups compared with standard errors. At the rupture site, p<0.0001; At the distant site, p<0.003.

PPROM =  Preterm, premature rupture of membranes (subjects between 24 and 34 weeks following confirmation of rupture of membranes by physical exam).

Preterm Labor  =  subjects between 24 and 34 weeks of gestation with spontaneous preterm labor.

Preterm, No Labor  =  subjects between 24 and 34 weeks of gestation delivered preterm without labor exposure.

Term  =  subjects between 37 and 41 weeks with labor.

Term, No Labor  =  subjects between 37 and 41 weeks without exposure to labor.

Rupture  =  sample of membrane collected at site of rupture.

Distant  =  sample of membrane collected from an area distant to rupture site, most commonly near the placenta.

Bacterial presence was not significantly associated with the administration of antibiotics or with the latency interval of time from membrane rupture to delivery. As expected, the mean bacterial count was significantly higher among those subjects with chorioamnionitis compared to those without at the both sites (Rupture: 38 vs. 23 bacteria, p = 0.025; Distant: 31 vs. 14 bacteria, p = 0.002).

Acknowledging the impact histologic infection had on bacterial presence, we repeated the analysis excluding subjects with histologic chorioamnionitis (n = 40, after removing subjects with chorioamnionitis) with similar trends noted. Among PPROM subjects without histopathologic infection, at both rupture site and distant site, the mean bacteria count per quadrant was significantly higher compared to preterm and term subjects (all p<0.0001 at both sites, Figure 2B).

**Figure 3:**
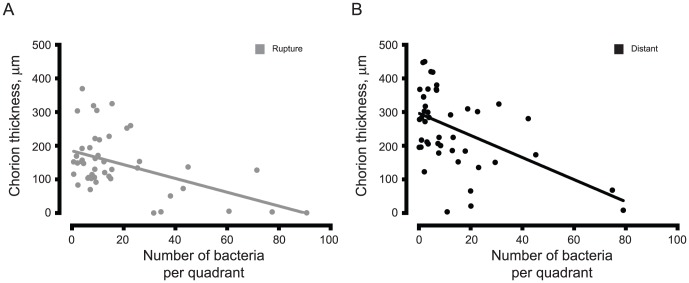
Correlation between Chorion Thickness and Bacterial Count Among all Samples by Membrane Location. Figure 3A is a plot demonstrating the inverse correlation noted between bacterial presence and chorion measurement at membrane rupture site among all samples examined. As chorion thins, bacterial counts increase. Figure 3B represents the inverse correlation between bacterial presence and chorion measurement at all “distant” membrane sites examined. Each dot signifies the correlation value for a single subject.

An inverse relationship between bacterial counts and chorion thinning was noted in PPROM subjects with or without chorioamnionitis. As an exploratory aim, we evaluated the correlation between chorion thinning and bacterial counts. Among all subjects, at both membrane sites sampled, an inverse relationship exists between bacterial presence and chorion measurement, such that as bacteria increases, the fetal chorion thins (All subjects, both sites, p<0.0001; rupture, p = 0.0006; distant, p = 0.0008). Figure 3. This finding persists excluding histologic infection at all sites sampled (p = 0.05).

Because qualitative evaluation of bacterial presence in the amnion varied significantly, the bacteria in the amnion and chorion of each sample were counted separately. Individual bacterial counts as well as the ratio of bacteria present in the amnion versus bacteria present in the chorion was created to understand the directionality of bacterial presence. The Amnion/Chorion (A/C) ratio was greater among PPROM subjects compared to their preterm and term cohorts, indicating more bacteria had penetrated the amnion in PPROM subjects. Actual number of bacteria seen in the membrane layers is shown in Table 7. This finding was most notable at rupture site and among those with histologic chorioamnionitis.

**Table 7:** Mean Number of Bacteria by Membrane Layer Among Clinical Groups and Amnion/Chorion Ratio.

	Rupture Site					Distant Site				
	Amnion Bacterial LS Mean (SE)	P value	Chorion Bacterial LS Mean (SE)	P value	Amnion/Chorion Ratio	Amnion Bacterial LS Mean (SE)	P value	Chorion Bacterial LS Mean (SE)	P value	Amnion/Chorion Ratio
PPROM	12 (1.5)	<0.0001	25 (2.2)	<0.0001	0.48	5 (0.8)	<0.0001	19 (1.7)	<0.0001	0.26
PTL	2 (0.5)		9 (0.8)		0.22	1 (0.3)		7 (1.1)		0.14
PTNL	1 (0.2)		8 (0.8)		0.13	1 (0.2)		5 (0.6)		0.20
TL	1 (0.3)		14 (6.2)		0.07	1 (0.2)		6 (1.6)		0.17
TNL	1 (0.2)		7 (0.7)		0.14	1 (0.2)		5 (0.7)		0.20

Values shown represent mean number of bacteria visualized within the amnion, the chorion, and the representative A/C ratio. Bacterial mean reporting includes standard errors and p values were <0.0001.

PPROM =  Preterm, premature rupture of membranes (subjects between 24 and 34 weeks following confirmation of rupture of membranes by physical exam).

Preterm Labor  =  subjects between 24 and 34 weeks of gestation with spontaneous preterm labor.

Preterm, No Labor  =  subjects between 24 and 34 weeks of gestation delivered preterm without labor exposure.

Term  =  subjects between 37 and 41 weeks with labor.

Term, No Labor  =  subjects between 37 and 41 weeks without exposure to labor.

Rupture  =  sample of membrane collected at site of rupture.

Distant  =  sample of membrane collected from an area distant to rupture site, most commonly near the placenta.

## Discussion

In this study, by examining precise sites within the same human fetal membrane, we found the fetal chorion layer to be differentially thinned at membrane rupture site as compared to more distant sites among all subjects suggesting that the chorion around the rupture site undergoes significant remodeling throughout gestation. In contrast, in the setting of PPROM, the chorion layer is thinner at all sites, or throughout the fetal membrane, indicating a more global, not localized, process is occurring. Surprisingly, this finding persists even in the absence of histopathologically confirmed infection. The chorion appears to be preferentially reduced in PPROM subjects throughout the membranes as compared to their preterm and term cohorts, implying that the chorion layer is primarily undergoing change.

Among women with PPROM, bacterial presence is greater at the rupture site with localization to the membranes overlying the cervix suggesting an ascending route for bacterial colonization. Interestingly, no sample was without bacterial fluorescence indicating that all fetal membrane samples had bacteria present irrespective of gestational age, labor, or membrane rupture. Bacterial presence was greatest among PPROM subjects, a finding that persisted regardless of gestational age, labor status, or infection. Overall, bacterial presence is inversely correlated with chorion thinning suggesting that the chorion is the layer that changes and that bacterial presence impacts this layer. In fact, regardless of infection, gestational age, or labor status, as the chorion thins bacterial presence increases. Although a causal relationship between bacterial presence, chorion remodeling, and membrane rupture remain unclear; it is plausible that bacterial presence leads to chorion thinning and ultimately PPROM.

Membrane rupture has been the focus of much research. Recently, the sequence of events leading to membrane rupture in full term membranes was evaluated mechanically. Rupture involves separation of the chorion from the amnion, fracture of the chorion, and then herniation and rupture of the amnion [22]. Our work suggests PPROM is unique and has its own pathologic process. The chorion thinning in PPROM described here suggests that some process, whether apoptosis, mechanical stretch, or chemical signaling [29] is at work globally in the membranes of women with PPROM. Prior work has demonstrated increased apoptosis in the fetal chorion of membrane samples with histologic chorioamnionitis and among samples with PPROM [24], [25] and [30], suggesting that the mechanical rupture of term membranes described by Arikat et al is only part of the process among women with PPROM [22]. We postulate that the chorion undergoes pathologic thinning (likely due to increased cellular apoptosis), which weakens the tensile strength or decreases the physical barrier and impedance to bacteria. Interestingly, the process of labor does not appear to impart changes in fetal membranes. The identification of bacteria in all fetal membrane tissues is consistent with the work of others [14], and implies that the bacterial colonization and species present are likely pivotal to understanding the pathologic process.

Identification of bacteria in preterm subjects without labor or histologic infection supports the hypothesis that bacteria may not be entirely pathogenic and not the lone cause of PPROM. Steel et al used the conserved prokaryotic 16 s ribosomal RNA sequence labeled with fluorescein to probe fetal membranes using fluorescence *in situ* hybridization (FISH) [14]. Bacteria were found in over 80% of PPROM patients, but were also present in fetal membranes of preterm deliveries and term deliveries with and without labor. They concluded that bacteria may be present in fetal membranes, but this alone is not sufficient to cause preterm labor and delivery[14]. Correspondingly, our data found similar bacterial counts among preterm and full term membranes, with and without labor exposure, again suggesting that regardless of gestational age or presence of labor, bacteria are present.

Identification of bacteria in term labor subjects is not surprising, but the absence of histologic chorioamnionitis among these membranes is intriguing. The fact that chorioamnionitis rates are higher in preterm labor compared to term labor despite nearly the same bacterial count also begs the question that some degree of tolerance/tissue inhibitors exist in term membranes. Prior studies have affirmed the finding of bacteria in at least one sample from their preterm labor and PPROM subjects [31], [32]. In 2009, Kim et al evaluated both amniotic fluid and fetal membranes from patients with intra-amniotic infection using both FISH and PCR [32]. They proposed a mechanism where discrete regions of the membranes are compromised and allow bacteria to traverse the membranes resulting in intra-amniotic infection. Little is known regarding discrete regions of the fetal membrane or of the changes occurring within the membranes in consequence or defense of bacterial presence, but our data suggests the chorion and its thinning may be the “battleground”.

Our study is unique in that it examined paired membrane samples from a known site of collection, obtained uniformly among all samples, from the clinical group of interest with comparison to well phenotypes with each serving as her own control. Describing bacteria in fetal membranes regardless of gestational age and correlating bacterial presence to changes in the fetal chorion within the same tissue sample provides novel information and potentially a unique perspective on bacterial presence versus pathogenesis in human fetal membranes. Our data reveals information on localizing bacteria, both in terms of proximity to rupture site, but also in terms of the fetal membrane layer. Specimen collection done prospectively and with relatively few investigators provided expeditious, reliable, and uniform tissue samples allowing interpretation of the localization data. Our dual sample collection method allowed examination of the variation within a fetal membrane sample. Our work is consistent with the work of others that have demonstrated a “zone of weakness” overlying the cervix among nonlabored, term, human fetal membranes [33], [34], [35]. What others noted in membrane strength disparities and morphologic evaluation over the cervix, we observed visually by functional chorion and choriodecidua measurements within the membrane, among all gestational ages, with and without exposure to labor. Furthermore, from our data, among PPROM subjects, properties similar to this “area of high morphologic change,” [34], exist throughout the fetal membranes, and not just overlying the cervix. The correlation with high bacterial presence at membrane rupture site provides insight into possible mechanisms.

There are several factors that could interfere with our stated aims and conclusions. Bacteria identified using FISH have not yet been correlated with PCR amplification and sequencing for species identification. From our data, there remains no clear elucidation of temporal sequence of events, although our data supports that of Steel, DiGuilo in suggesting that bacteria are present throughout membranes with our data purporting this bacterial presence exists regardless of route of delivery. [36], [37]. As with any sample collected following vaginal delivery, bacteria detected in these samples may represent contamination of the tissue during passage through the vagina, and thus next steps will focus on identification of these bacteria. Identification of the chorion and bacteria that would be in that chorion may be underestimated due to tissue friability in processing both during sample collection and slide preparation. Although tissue processing may impact reproducibility, we chose to overcome this limitation by (1) identifying the trophoblast cells of human fetal chorion via staining because we were interested in measuring the presence of only the trophoblast layer and (2) by repeated measurements by the same investigator. Trophoblast cells in the chorion are the most metabolically active making them the most germane to study [20], [38]. Despite subject variability and tissue processing changes, the large sample size and duplicated measurements allowed us to determine a significant difference by clinical phenotype. Finally, all images were measured and scored for fluorescence at least twice with clinical identification blinded to the reviewer (primary author). Another author (CER) scored fluorescence in over half of the samples for confirmation. The slides were all reviewed by the same staff pathologist, providing reliable designation of “chorioamnionitis” or not.

In conclusion, we describe differences within human fetal membranes by proximity to the rupture site/area overlying the cervix. We detected chorion thinning and bacterial presence in the rupture site. In PPROM, the chorion is significantly thinned and bacterial presence is significantly higher regardless of labor exposure, gestational age, or presence of histologic chorioamnionitis compared to other clinical phenotypes. It remains unclear whether chorion thinning is the cause or consequence of PPROM, but thinned chorion certainly appears to play an important role in membrane integrity. The goal of this work is to advance the understanding of PPROM. Future work should focus on the identification of specific bacterial species within fetal membranes from all clinical phenotypes. Identification of specific bacterial species may advance our understanding of the role of bacterial presence and lead to potential targeted therapeutic interventions.

## Supporting Information

Figure S1 **Specimen collection.** Membrane rupture and distant sites were identified and a strip of membrane was collected. The membrane strip was rolled, stabilized, sectioned, and then formalin fixed and paraffin embedded.
(TIFF)

Figure S2 **Immunohistochemistry.** Sample slide. Figure demonstrates that each slide contained four representative sections of the rolled membrane. Figure also demonstrates stained trophoblast layer of the chorion and cuboidal cells of the amnion. Images were obtained at 10× magnification from four separate areas of each membrane roll.
(TIFF)

Figure S3 **Immunohistochemistry.** Viewed at 10× magnification, stained fetal chorion from membrane rupture is shown. Sample images demonstrate membrane “quadrants” and how chorion thickness compared by Clinical Group: Term, No Labor is compared to PPROM.
(TIFF)

Figure S4 **Fluorescent in situ hybridization (FISH) for bacterial DNA.** Slides were evaluated under appropriate UV wavelength with the Zeiss Axio Observer at 10X magnification. Images were captured via digital photography using three wavelengths for three color channels: blue (DAPI), red (probe), and green (autofluorescence), and then overlay. A composite overlay image was obtained by combining the three channels resulting in nuclei appearing blue, bacteria appearing red, and background tissue autofluorescence appearing yellow (red plus green) or green. Additional 40X image included demonstrating bacteria around an individual cell nuclei.
(TIFF)
