# Supplementary figures and images for: Bacteria Localization and Chorion Thinning among Preterm Premature Rupture of Membranes
Source: PLoS One. 2014 Jan 8;9(1):e83338. doi: 10.1371/journal.pone.0083338 (PMC3885429; doi:10.1371/journal.pone.0083338)

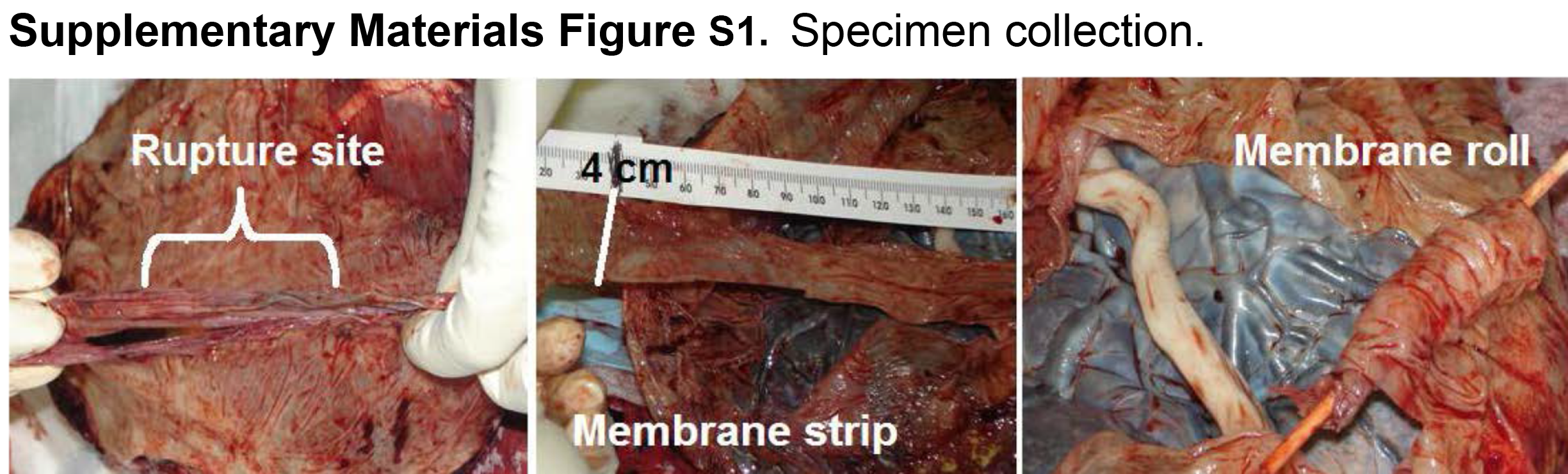

Supplement: Figure S1 — Specimen collection. Membrane rupture and distant sites were identified and a strip of membrane was collected. The membrane strip was rolled, stabilized, sectioned, and then formalin fixed and paraffin embedded. (TIFF) [file pone.0083338.s001.tiff]

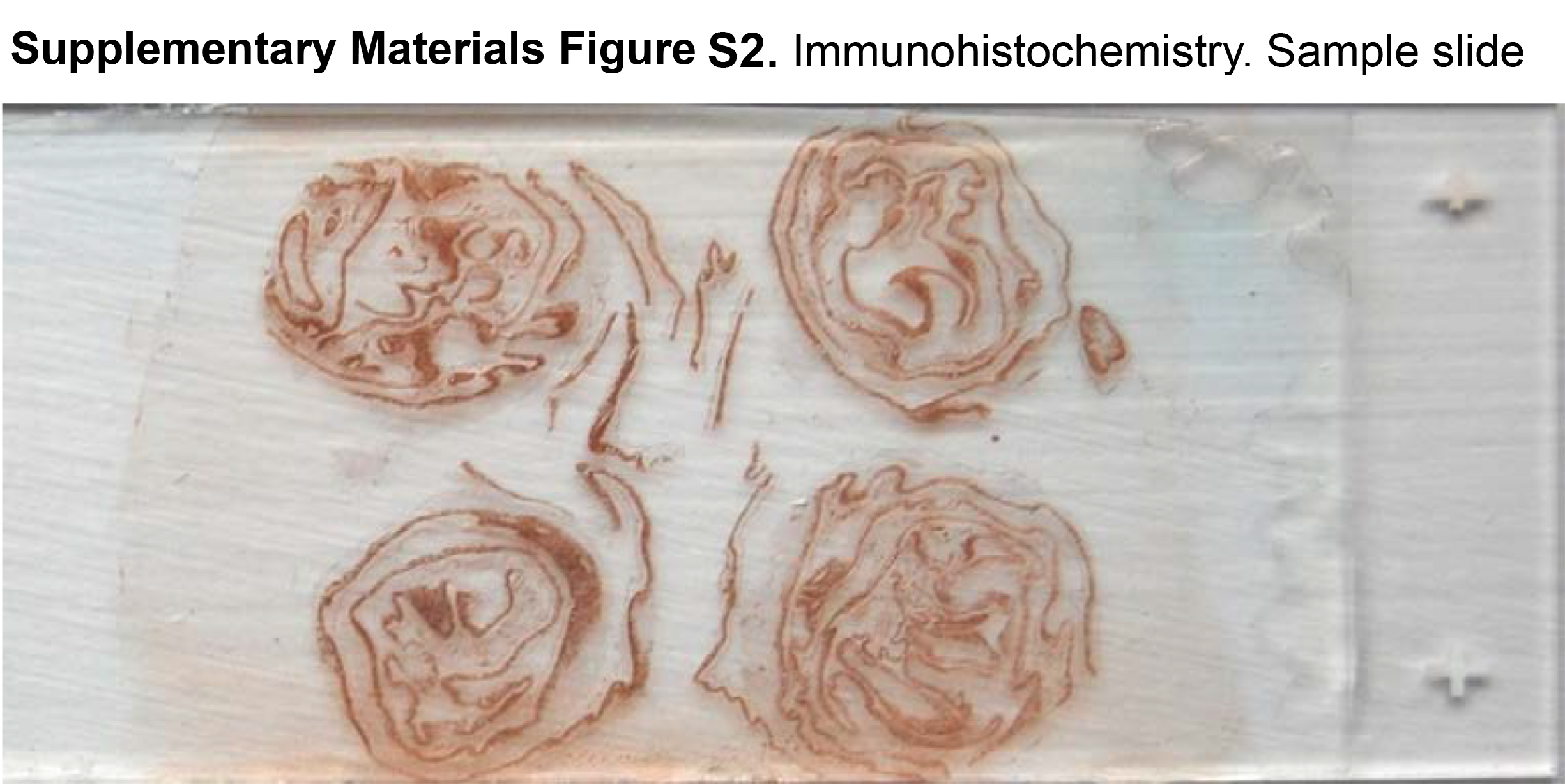

Supplement: Figure S2 — Immunohistochemistry. Sample slide. Figure demonstrates that each slide contained four representative sections of the rolled membrane. Figure also demonstrates stained trophoblast layer of the chorion and cuboidal cells of the amnion. Images were obtained at 10× magnification from four separate areas of each membrane roll. (TIFF) [file pone.0083338.s002.tiff]

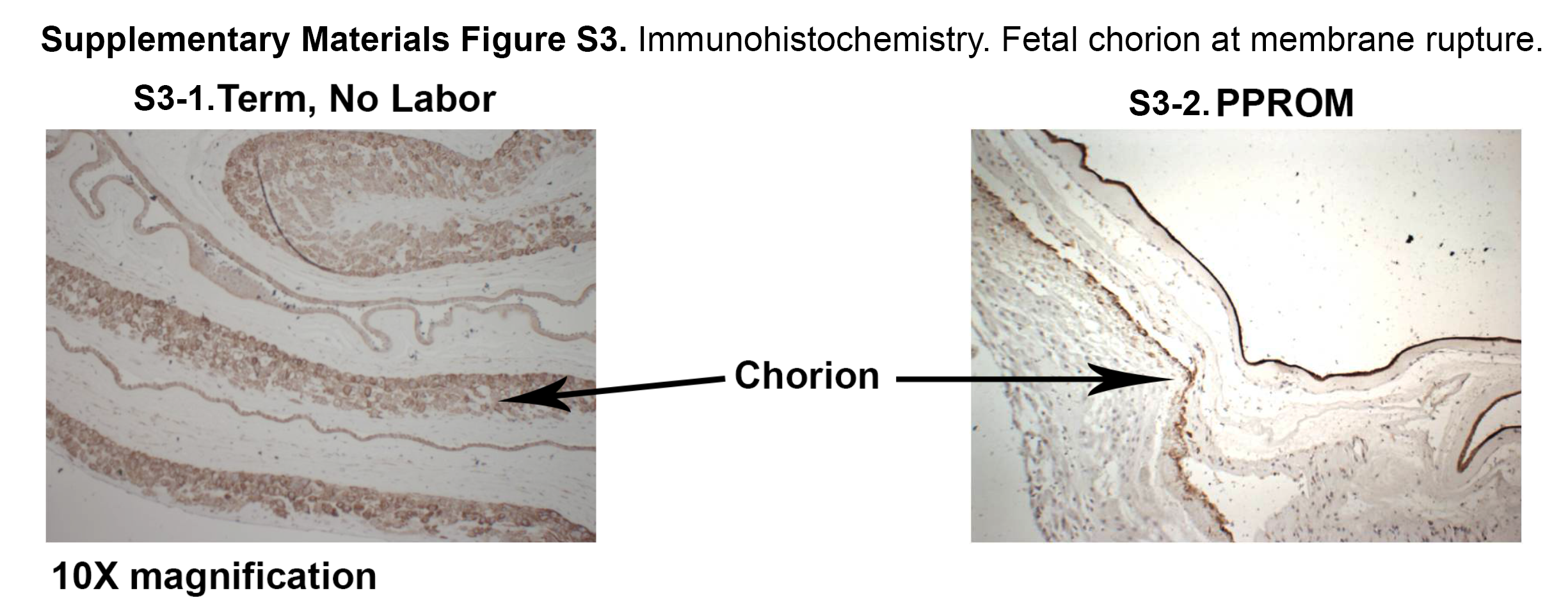

Supplement: Figure S3 — Immunohistochemistry. Viewed at 10× magnification, stained fetal chorion from membrane rupture is shown. Sample images demonstrate membrane “quadrants” and how chorion thickness compared by Clinical Group: Term, No Labor is compared to PPROM. (TIFF) [file pone.0083338.s003.tiff]

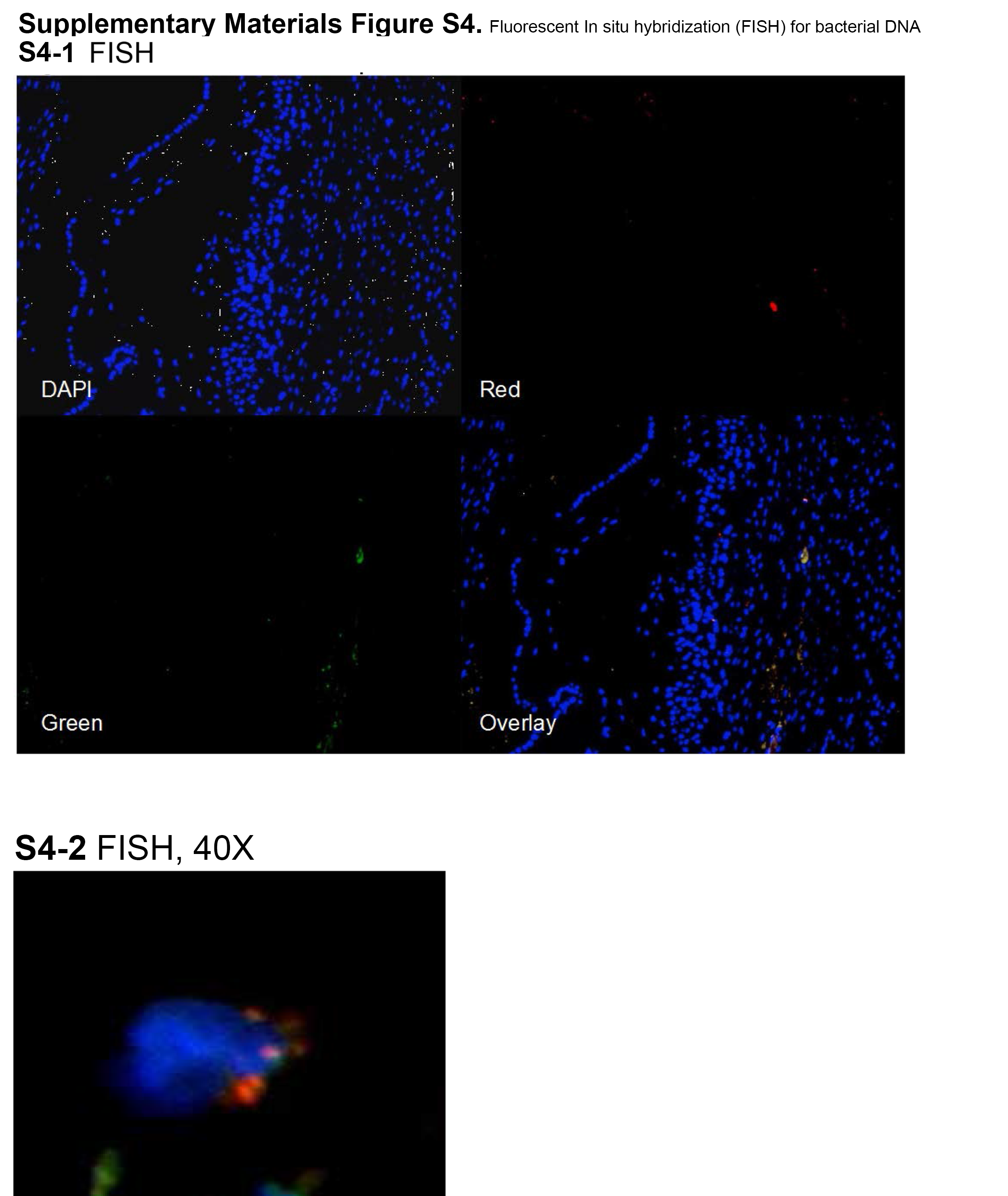

Supplement: Figure S4 — Fluorescent in situ hybridization (FISH) for bacterial DNA. Slides were evaluated under appropriate UV wavelength with the Zeiss Axio Observer at 10X magnification. Images were captured via digital photography using three wavelengths for three color channels: blue (DAPI), red (probe), and green (autofluorescence), and then overlay. A composite overlay image was obtained by combining the three channels resulting in nuclei appearing blue, bacteria appearing red, and background tissue autofluorescence appearing yellow (red plus green) or green. Additional 40X image included demonstrating bacteria around an individual cell nuclei. (TIFF) [file pone.0083338.s004.tiff]
